# Individualized analysis reveals CpG sites with methylation aberrations in almost all lung adenocarcinoma tissues

**DOI:** 10.1186/s12967-017-1122-y

**Published:** 2017-02-08

**Authors:** Haidan Yan, Qingzhou Guan, Jun He, Yunqing Lin, Juan Zhang, Hongdong Li, Huaping Liu, Yunyan Gu, Zheng Guo, Fei He

**Affiliations:** 1 Department of Systems Biology, College of Bioinformatics Science and Technology, Harbin Medical University, Harbin, 150086 China; 2 Key Laboratory of Ministry of Education for Gastrointestinal Cancer, Department of Bioinformatics, Fujian Medical University, Fuzhou, 350001 China; 3 Key Laboratory of Ministry of Education for Gastrointestinal Cancer, Department of Epidemiology and Health Statistics, School of Public Health, Fujian Medical University, Fuzhou, 350001 China

**Keywords:** Lung adenocarcinoma, DNA methylation, Relative methylation level orderings, Differentially methylated CpG sites

## Abstract

**Background:**

Due to the heterogeneity of cancer, identifying differentially methylated (DM) CpG sites between a set of cancer samples and a set of normal samples cannot tell us which patients have methylation aberrations in a particular DM CpG site.

**Methods:**

We firstly showed that the relative methylation-level orderings (RMOs) of CpG sites within individual normal lung tissues are highly stable but widely disrupted in lung adenocarcinoma tissues. This finding provides the basis of using the RankComp algorithm, previously developed for differential gene expression analysis at the individual level, to identify DM CpG sites in each cancer tissue compared with its own normal state. Briefly, through comparing with the highly stable normal RMOs predetermined in a large collection of samples for normal lung tissues, the algorithm finds those CpG sites whose hyper- or hypo-methylations may lead to the disrupted RMOs of CpG site pairs within a disease sample based on Fisher’s exact test.

**Results:**

Evaluated in 59 lung adenocarcinoma tissues with paired adjacent normal tissues, RankComp reached an average precision of 94.26% for individual-level DM CpG sites. Then, after identifying DM CpG sites in each of the 539 lung adenocarcinoma samples from TCGA, we found five and 44 CpG sites hypermethylated and hypomethylated in above 90% of the disease samples, respectively. These findings were validated in 140 publicly available and eight additionally measured paired cancer-normal samples. Gene expression analysis revealed that four of the five genes, HOXA9, TAL1, ATP8A2, ENG and SPARCL1, each harboring one of the five frequently hypermethylated CpG sites within its promoters, were also frequently down-regulated in lung adenocarcinoma.

**Conclusions:**

The common DNA methylation aberrations in lung adenocarcinoma tissues may be important for lung adenocarcinoma diagnosis and therapy.

**Electronic supplementary material:**

The online version of this article (doi:10.1186/s12967-017-1122-y) contains supplementary material, which is available to authorized users.

## Background

The incidence of lung adenocarcinoma is increasing worldwide. It is widely recognized that lung adenocarcinoma, like other cancers, has different molecular subtypes with different prognoses [1, 2]. In cancer genomes, the frequencies of somatic mutations and copy number variations are usually very low [3, 4], forming a major barrier for cancer diagnosis and therapy. On the other hand, it is also well known that cancers are commonly characterized with cancer hallmarks [5, 6], and tremendous efforts have been made to identify common biomarkers at the level of pathways or gene modules. Especially, mutually exclusive analyses of somatic mutation and/or copy number variation have been tried to identify some driver genes that could jointly explain a large fraction samples of a cancer type to provide potential targets of medicine [7, 8]. However, a set of mutually exclusive genes or gene modules as a cancer diagnosis and therapy maker might be difficult for clinical applications. In contrast to genomic aberrations, DNA methylation aberrations are widespread in cancer genomes. Therefore, it would be interesting to analyze whether there are epigenomic aberrations appearing in almost all patients of a cancer such as lung adenocarcinoma.

Currently, genome-wide DNA methylation profiles are widely applied to identify population-level differentially methylated (DM) CpG sites in cancer tissues compared with normal controls using various statistical methods such as the Shannon entropy based method QDMR [9], empirical Bayes model DiffVar [10] and *T* test [11]. However, the inter-individual heterogeneity of DM CpG sites was ignored in these approaches. Thus a novel computational tool is needed to detect which patients have methylation aberrations in a particular CpG site. To tackle this difficulty, some previous works discretized DNA methylation states of CpG sites in a cancer sample through comparing with the average methylation level in a set of normal samples [12, 13]. However, because the DNA methylation levels of CpG sites vary across individuals in a healthy population, this approach may easily be affected by biological variations.

Recently, we have reported an interesting biological phenomenon that the within-sample relative expression orderings of genes within a particular type of normal tissues are highly stable but widely disrupted in the corresponding cancer tissues [14]. Based on this finding, we have developed an algorithm, named RankComp, to identify genes differentially expressed in each individual cancer tissue sample by finding those genes whose up- or down-regulations may lead to the disrupted relative expression orderings of genes within this disease sample in comparison with the stable normal background [14]. Importantly, the stable normal background of the relative expression orderings of genes within a particular type of normal tissues can be predetermined in accumulated normal samples previously measured by different laboratories [14]. Thus, it would be of interest to evaluate whether the within-sample relative methylation-level orderings (RMOs) of CpG sites are also highly stable in a particular type of normal tissues but widely disrupted in the corresponding cancer tissues. If this biological phenomenon does exist, then it would be possible to apply the RankComp algorithm to detect DM CpG sites for each cancer tissue compared with its own previously (usually unknown) normal status.

In this study, through the analysis of multiple methylation datasets for normal lung tissues, we firstly revealed an interesting biological phenomenon that the RMOs of CpG sites within different samples of normal lung tissues are highly stable but widely reversed in the cancer tissues. Based on this finding, we showed that the RankComp algorithm can accurately detect DM CpG sites in individual cancer samples from DNA methylation data for cancer samples alone. Then, RankComp was applied to identify DM CpG sites for each of the 539 lung adenocarcinoma samples from The Cancer Genome Atlas (TCGA). Many CpG sites with methylation aberrations in above 90% of lung adenocarcinoma tissues were found and validated in 140 publicly available and eight additionally measured paired cancer-normal samples. Gene expression analysis revealed that four of the five genes, HOXA9, TAL1, ATP8A2, ENG and SPARCL1, each harboring one of the five frequently hypermethylated CpG sites within its promoters, were also frequently down-regulated in lung adenocarcinoma.

## Methods

### Data and preprocessing

DNA methylation profiles for lung tissues were collected from the Gene Expression Omnibus (GEO) [15] database and The Cancer Genome Atlas data portal (http://tcga-data.nci.nih.gov/tcga/). We used a dataset (GSE32861) of DNA methylation profiles for paired cancer and adjacent normal samples to evaluate the performance of RankComp (Table 1). Except the paired cancer-normal datasets, the other DNA methylation profiles described in Table 1 were used to evaluate the RMOs of CpG sites in normal and cancer tissues. The DNA methylation profiles of 539 samples of lung adenocarcinoma were selected from TCGA for application analysis.

**Table 1:** The DNA methylation profiles analyzed in this study

Dataset	Normal	Tumor	Platform
GSE62948	28	28	27K
GSE32866	27	28	27K
GSE52401	244	–	450K
TCGA	24	109	27K
TCGA	32	430	450K
GSE32861^a^	59	59	27K

^a^Represents the paired cancer-normal samples used to evaluate the performance of Rankcomp

The DNA methylation data was measured with Illumina Human Methylation 27 Beadchip (27K array) and Illumina Human Methylation 450 Beadchip (450K array). We focused on analyzing the 25,978 CpG sites measured by both 27 and 450K arrays. Using methylated signal intensity (M) and unmethylated signal intensity (U), the DNA methylation level of each probe was calculated by M/(U + M + 100) [16]. The probes were annotated to genes according to the annotation table of 27K platform.

### KEGG pathways

Data of 234 pathways covering 5981 unique genes was downloaded from the Kyoto Encyclopedia of Genes and Genomes (KEGG) (Release 58.0) [17] for pathway enrichment analysis.

### Reproducibility analysis of the stable RMOs of CpG sites in normal tissues

The RMO of two CpG sites, A and B, was denoted as A > B or A < B if the site A had a higher or lower methylation level than the site B. The RMO of two CpG sites was defined as stable in normal tissues if the same RMO existed in at least 99% of the samples, allowing 1% detection error rate.

For a particular type of normal tissues, we respectively detected two lists of stable CpG site pairs in two independent datasets to evaluate the reproducibility of the stable CpG site pairs. The concordance score of the two lists of stable CpG site pairs was calculated as *s/k*. *k* was the number of the stable CpG site pairs shared by the two lists, among which *s* pairs had the same RMO patterns (A > B or A < B) in the two lists. The probability of a concordance score *s/k* observed by chance was calculated according to the cumulative binomial distribution model [18].

1 $$ P = 1 - \sum\limits_{i = 0}^{s - 1} {\left( {\begin{array}{*{20}c} k \\ i \\ \end{array} } \right)} \left( {P_{e} } \right)^{i} \left( {1 - P_{e} } \right)^{k - 1} $$

where *Pe* (*Pe* = 0.5) is the probability of a pair of CpG sites having the same RMO in two lists by chance.

### Reproducibility analysis of the reversal RMOs of CpG sites in cancer tissues

For stable RMOs in normal samples, we applied Fisher’s exact test to detect gene pairs with significantly higher frequencies of reversal RMOs in cancer samples than what expected by random chance using two independent datasets, respectively, defined as reversal CpG site pairs. The p-values were adjusted using the Benjamini–Hochberg procedure [19]. The concordance of two lists of reversal CpG site pairs identified from two independent datasets was also evaluated by the cumulative binomial distribution model as described above.

### The RankComp algorithm for individualized differential methylation analysis

Then, we used RankComp to identify differentially methylated CpG sites in a given cancer sample in comparison with its previously normal state, based on the highly stable CpG site pairs with consistent RMOs in at least 99% of previously accumulated normal samples measured by different laboratories. The detail of the RankComp algorithm was described in Wang et al. [14]. Briefly, for each cancer sample, CpG site pairs with reversal ordering in comparison with their stable ordering in normal samples were determined as reversal CpG site pairs by RankComp. Then, the Fisher’s exact test was used to determine whether a CpG site *Ci* was differentially methylated in a given cancer sample by testing the null hypothesis that the proportion of reversal CpG site pairs supporting the hypermethylation of *Ci* was equal to the proportion of reversal CpG site pairs supporting the hypomethylation of *Ci*. For a given CpG site *Ci*, if its ordering was stably lower (or higher) than that of CpG site *Cj* in normal samples but this ordering was reversed in a cancer sample, then this reversal CpG site pair could support hypermethylation (or hypomethylation) of *Ci* in this sample. If *Ci* itself is not changed in methylation level, the effect of the methylation changes of other CpG sites on the upward or downward shift in the rank of *Ci* is assumed to be a random event.

### Performance evaluation of RankComp

Methylation profiles for paired cancer and adjacent normal tissues were downloaded from TCGA and GEO to evaluate the performance of RankComp. We used RankComp to detect DM CpG sites in individual cancer samples using DNA methylation data on cancer samples alone. After identifying DM CpG sites for each cancer sample, we evaluated the precision of DM CpG sites identified for this cancer sample using the observed methylation level differences (hyper- or hypomethylation) between the cancer sample and its paired adjacent normal sample as the golden standard. The underlying assumption of this evaluation is that the previously normal state of a cancer tissue could be approximately represented by the adjacent normal tissue of the cancer tissue. For a cancer sample, if the aberration states of DM CpG sites identified by RankComp are consistent with the golden standard, then they are defined as true positives (TP); otherwise, false positives (FP). The precision is calculated as positive predictive value: TP/(TP + FP).

To ensure the association between the individualized CpG sites and cancer, the precision analysis for each cancer sample was restricted to the CpG sites that were found to be differentially methylated at the population-level. Thus, we applied T-test to detect population-level DM CpG sites between cancer samples and normal samples using two independent datasets for each cancer, respectively. The Benjamini–Hochberg procedure was used to control the false discovery rate (FDR). The concordance score between the two lists of DM CpG sites and its statistical significance were calculated by the same method as described above. We defined the DM CpG sites that were consistently detected from the two independent datasets as the population-level DM CpG sites for each cancer.

### DNA methylation and gene expression profiling

Eight paired samples of lung adenocarcinoma tissues and adjacent normal tissues were used for DNA methylation and gene expression detection. Fresh frozen cancer tissue samples were obtained from surgically removed lung specimens. Tumor samples were macro dissected to ensure the purity of the tumor. From the same patient, adjacent normal tissue samples were collected from resected, located approximately 3.5–5 cm away from the tumor site. All samples were collected from the operating room immediately after surgical resection. Samples were fresh frozen and were shipped on ice for subsequent DNA extraction and RNA extraction. This study was approved by the institutional review boards of all participating institutions, and written consent forms were obtained from all participants.

DNA was extracted from frozen tissue samples using the Qiagen^®^ Genomic DNA Mini Kit, as described by the manufacturer. DNA quantification was performed using a NanoDrop 2000 UV–Vis Spectrophotometer (Thermo). The bisulfite conversion of DNA was conducted using the Zymo bisulfite gold kit. The Infinium Methylation 450K assay was performed according to Illumina’s standard protocol. Processed methylation chips were scanned using an iScan reader (Illumina). All samples were processed at the same time to avoid chip-to-chip variation. Infinium Methylation data were processed using the Methylation Module of the GenomeStudio software package (v. 2011.1). For quality control, methylation measures with a detection p-value >0.05 were removed. The data was initially normalized using internal controls in the GenomeStudio software and has been submitted to GEO (GSE85845).

Total RNA was extracted using Trizol reagent (Invitrogen) according to the manufacture’s protocol. The purity and concentration of RNA was determined by Nano Drop ND-1000 spectrophotometer according to OD260/280 reading. Each time point has three replicates. Total RNAs were hybridized using mRNA + lncRNA Human Gene Expression Microarray V4.0 (4 × 180K format, CapitalBio Corp, Beijing, China), which contains probes interrogating about 34,235 human mRNAs assembled from databases such as UCSC [20], RefSeq [21] and Ensembl [22]. The gene expression profiles have been submitted to GEO (GSE85841).

## Results

### Highly stable RMOs of CpG sites in normal lung tissues are widely disrupted in cancer tissues

We collected a total of 355 samples for normal lung tissues, including 79 samples derived from three datasets assayed by the 27K array and 276 samples derived from two datasets assayed by the 450K array (Table 1). Here, we only analyzed the CpG sites assayed by both the 27 and 450K arrays.

**Fig. 1:**
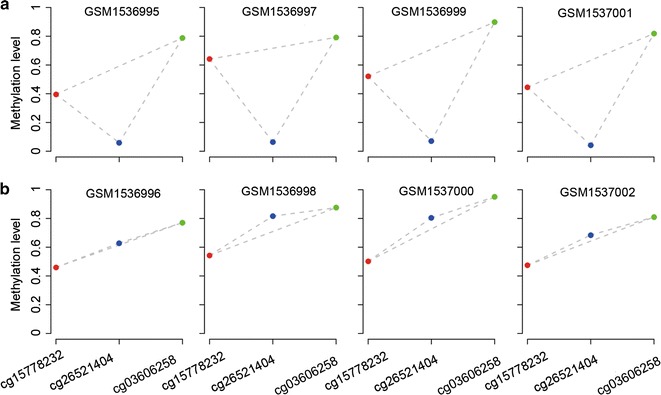
An example of three CpG sites with pairwise stable RMOs in normal samples (**a**) and reversal RMOs in cancer samples (**b**). Four paired cancer-normal samples from GSE32866 were used to show this biological phenomenon. *Red*, *blue* and *green* points represent cg15778232, cg26521404 and cg03606258 CpG sites, respectively

We defined two CpG sites as a stable CpG site pair if the two CpG sites had identical RMO in at least 99% of the normal lung tissue samples. Accordingly, a list of 229,037,151 stable CpG site pairs was identified in the 79 normal lung tissue samples assayed by the 27K array, and a shorter list of 173,949,484 stable CpG site pairs was identified in the 276 normal lung tissue samples assayed by the 450K array (Additional file 1: Table S1). Notably, 90.62% of the stable CpG site pairs in the shorter list were included in the longer list and 99.75% of the overlapped CpG site pairs had the same RMOs in the two sets of the normal lung tissue samples (binomial test, *p* < 2.2 × 10–16). The stable CpG site pairs involved all the measured CpG sites. These results suggest that the highly stable within-sample RMOs of CpG sites in normal lung tissues can be reproducibly detected across samples measured with different platforms. As exemplified in Fig. 1a, although the DNA methylation levels of cg15778232, cg26521404 and cg03606258 CpG sites varied greatly across different normal samples in the GSE32866 dataset, their RMOs (cg15778232 > cg26521404, cg26521404 < cg03606258 and cg15778232 < cg03606258) were highly stable across the normal samples.

Among the CpG site pairs with stable RMOs in normal lung tissue, we found 8,615,527 and 37,815,005 CpG site pairs with significantly frequent reversal RMOs in the 165 and 430 lung cancer samples assayed by the 27K array and 450K array (Table 1) (FDR < 0.05, Fisher’s exact test), respectively. As exemplified in Fig. 1b, the RMO of cg15778232 and cg26521404 was widely reversed in the lung cancer samples (GSE32866), caused by the methylation level increase of cg26521404. The reversal CpG site pairs involved all the measured CpG sites and averagely a CpG site participated in more than four thousands of reversal CpG site pairs. These results suggest that the landscape of stable CpG site pairs in normal lung tissues is widely disrupted in lung cancer tissues.

### Performance evaluation of RankComp

Here, we firstly determined DM CpG sites associated with lung cancer at the population-level. From two independent datasets, GSE62948 and GSE32866, 6,515 and 5,451 DM CpG sites in lung cancer were detected (*T*-test, FDR < 0.05) (Additional file 2: Table S2). The two lists of DM CpG sites had 4,159 overlaps and all of them had the same hypermethylation or hypomethylation states in the two datasets with the concordance score being 100% (binomial test, *p* < 2.2 × 10–16). These 4159 reproducible DM CpG sites were defined as the population-level DM CpG sites for lung cancer.

**Fig. 2:**
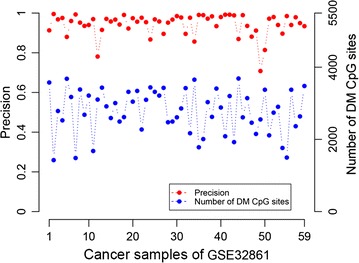
The precision and the number of DM CpG sites detected by RankComp for each of the 59 lung cancer samples from GSE32861

Then, we used an independent dataset, GSE32861 with 59 paired cancer-normal lung samples, to evaluate the performance of RankComp in individualizing the above defined population-level DM CpG sites. Here, we detected DM CpG sites for each disease sample without using any of the methylation data of its paired adjacent normal sample. The paired adjacent normal sample of a disease sample was used for performance evaluation only: the observed methylation level differences of the DM CpG sites between the cancer sample and its adjacent normal sample were taken as the golden standard. Based on the predetermined stable CpG site pairs in the accumulated 355 normal lung tissue samples, with FDR < 0.01, averagely 2777 DM CpG sites per disease sample were identified and the average of the precisions of all samples was 94.26%. However, as shown in Fig. 2, the precision for a sample (GSM813269) was only 70.89%, which may be caused by the quality of its paired normal tissue. In fact, the Spearman’s correlation coefficient between the DNA methylation levels of this normal tissue and any of the other 58 normal tissues was lower than 0.72, while the Spearman’s correlation coefficient was larger than 0.88 between every two of the other 58 normal tissues.

The above results suggested that RankComp can accurately find DM CpGs in individual lung cancer tissues compared with their own previous normal state approximately represented by their paired adjacent normal tissues.

### The CpG sites with methylation aberrations in above 90% lung adenocarcinoma tissues

Then, we used RankComp to identify DM CpG sites for each of the 539 lung adenocarcinoma samples from TCGA. The results showed that 83.64% of DNA methylation aberrations appeared in less than 80% of the cancer samples, reflecting the heterogeneity nature of cancer. Interestingly, we found five and 44 CpG sites that were hypermethylated and hypomethylated, respectively, in more than 90% of the 539 cancer samples (Additional file 3: Table S3). In the following text, we focused on validating these CpG sites with extremely high frequencies of methylation alternations in cancer tissues.

Firstly, we validated these CpG sites using 140 paired samples of cancer-normal tissues collected from three datasets (GSE32861, TCGA and GSE62948, Additional file 4: Table S4). As shown in Table 2, in more than 95% of the 140 paired samples, the methylation levels of the five CpG sites with high hypermethylation frequencies were higher in the cancer tissues than in the corresponding adjacent normal tissues. Similarly, in more than 90% of the 140 paired samples, 43 of the 44 hypomethylated CpG sites had lower methylation levels in the cancer tissues than in the corresponding adjacent normal tissues (Additional file 5: Table S5).

**Table 2:** The hypermethylation frequencies of five CpG sites in disease samples

CpG site	Gene symble	Frequency^1^ (%)	Frequency^2^ (%)
cg05050341	ENG	97.86	100
cg12111714	ATP8A2	97.86	100
cg19466563	SPARCL1	98.57	87.50
cg19797376	TAL1	98.57	100
cg26521404	HOXA9	98.57	100

For a gene corresponding to a CpG site, Frequency^1^ and Frequency^2^ represent the hypermethylation frequencies in the 140 publicly available and eight additionally measured paired cancer-normal samples, resepectively

To further validate these CpG sites with extremely high frequencies of methylation alternations, we measured DNA methylation profiles for eight paired cancer-normal lung tissues using Illumina Human Methylation 450 Beadchip. Four of the five frequently hypermethylated CpG sites were hypermethylated in all the eight paired cancer-normal samples and the left one CpG site was hypermethylated in seven paired cancer-normal samples (Table 2). For the 44 hypomethylated CpG sites with high frequencies, 39 CpG sites were validated in at least seven paired cancer-normal samples (Additional file 5: Table S5).

### Genes deregulated by the frequently hypermethylated CpG sites

Because it is well known that DNA hypomethylation is weakly correlated with up-regulation of gene expression [23], we focused on analyzing the five hypermethylated CpG sites with above 90% frequencies in lung adenocarcinoma tissues. We defined a gene as a hypermethylated gene in a disease sample if at least one CpG site within its promoter region was identified as a hypermethylated and no CpG site was identified as hypomethylated. Accordingly, we found five genes (HOXA9, TAL1, ATP8A2, ENG and SPARCL1) corresponding to the five CpG sites had above 90% hypermethylation frequencies in the 539 lung adenocarcinoma samples from TCGA.

Then, we investigated the expressions of the five genes using 82 paired cancer-normal samples with gene expression profiles (GSE32867 and TCGA, Additional file 6: Table S6). Here, a gene was defined as down-regulated in the cancer sample if its expression level in the cancer sample was lower than that in the corresponding adjacent normal sample. We found that SPARCL1, ENG, TAL1, ATP8A2 and HOXA9 were down-regulated in 99, 94, 89, 65 and 49% of the 82 cancer samples (Table 3). Thus, at least three genes, SPARCL1, ENG and TAL1, might be frequently and strongly silenced by the frequently hypermethylated CpG sites within their promoter regions. To further validate the results for these three genes, we measured gene expression profiles for eight paired cancer-normal lung tissues using microarray. The gene expression levels of SPARCL1 and TAL1 were down-regulated in all the eight cancer tissues compared with their paired adjacent normal tissues, while ENG was down-regulated in six of the eight cancer tissues (Table 3).

**Table 3:** Down-deregulation frequencies of the five genes regulated by the five frequently hypermethylated CpG sites

CpG site	Gene symble	Frequency^1^ (%)	Frequency^2^ (%)
cg05050341	ENG	93.90	75.00
cg12111714	ATP8A2	64.63	62.50
cg19466563	SPARCL1	98.78	100
cg19797376	TAL1	89.02	100
cg26521404	HOXA9	48.78	50.00

For a gene corresponding to a CpG site, Frequency^1^ and Frequency^2^ represent the down-regulation frequencies of the gene in the 82 publicly available and eight additionally measured paired cancer-normal samples, resepectively

The above results suggested that SPARCL1, ENG and TAL1 could be potential tumor suppressors of lung adenocarcinoma and thus could be drug targets or diagnostic biomarkers for lung adenocarcinoma. ENG is a candidate tumor-suppressor gene of esophageal squamous cell carcinoma [24]. It has been found that the silencing of ENG by hypermethylation in the promoter region could be reactivated by demethylation [25] that can reduce colony formation efficiency and suppress invasion efficiency and tumorigenicity of cancer cells [24]. Thus, the drug target potential of ENG for lung adenocarcinoma deserves further studies. SPARCL1, a known tumor suppressor of colon cancer [26], is an anti-adhesive extracellular matrix gene with anti-proliferative effects mediated through cell–cell adhesion [27, 28]. TAL1 is a member of the basic helix-loop-helix family of transcription factors and its downregulation could suppress the activity of the tumor suppressor TGF-β in the early phase of tumor through downregulating KDR expression [29]. Thus, SPARCL1 and TAL1 might also be important tumor suppressors of lung adenocarcinoma and it would be interesting to study whether their silencing could be reactivated by demethylation and whether the reactivation could suppress cancer cells.

## Discussion

The inter-individual heterogeneity of cancer forms a major barrier for cancer diagnosis and therapy. Thus, it would be interesting to find common molecular aberrations appearing in almost all patients of a cancer such as lung adenocarcinoma. In this work, we have revealed an interesting biological phenomenon that the within-sample RMOs of CpG sites remain highly stable in normal lung tissues. This might be an epigenetic mechanism to keep genes functioning concertedly and robustly in the normal lung tissues in the presence of various perturbations. Another interesting biological phenomenon revealed in this study is that the stable RMOs in normal tissues are widely disrupted in the corresponding cancer tissues, reflecting the nature of cancer as a systems disease. These intrinsic biological phenomena provide the basis for identifying DM CpG sites for an individual cancer sample by exploiting the widely disrupted epigenetic landscape within this individual disease sample. In fact, our analyses showed that the rank-based RankComp algorithm can accurately detect DM CpG sites at the individual-level, providing us a novel tool to dissect the inter-individual heterogeneity of cancer patients. Then, through the individual-level analysis of DM CpG sites in 539 lung adenocarcinoma samples, we found five and 44 CpG sites hypermethylated and hypomethylated in above 90% of the disease samples, respectively. These findings were further validated in 140 publicly available and eight additionally measured paired cancer-normal samples. These common DNA methylation aberrations found in lung adenocarcinoma tissues may be important for lung adenocarcinoma diagnosis and therapy. Especially, we found three genes (SPARCL1, ENG and TAL1) harboring frequently hypermethylated CpG sites within their promoters were also frequently down-regulated in lung adenocarcinoma. The results suggested that these genes might function as tumor suppressors and thus might be drug targets or diagnostic biomarkers of lung adenocarcinoma.

In addition, after identifying DM genes for a disease sample, we could detect pathways significantly enriched with hypermethylated and hypomethylated genes respectively for this disease sample. The application of the individual-level pathway analysis to 539 samples of lung adenocarcinoma revealed that the neuroactive ligand-receptor interaction pathway, known to be involved in cancer development [30, 31], was significant in 96.48% of the 539 samples, while the calcium signaling pathway, also known to be involved in cancer [32–34], was significant in 88.87% of the 539 samples (FDR < 0.05, Additional file 7: Figure S1). Therefore, these pathways significantly enriched with DM genes in almost all adenocarcinoma tissues deserve our future investigation.

Notably, the majority of the DNA methylation aberrations are not universally appearing in patients of lung adenocarcinoma. For example, 83.64% of the CpG sites with DNA methylation aberrations appeared in less than 80% of all the 539 lung adenocarcinoma tissues from TCGA, reflecting the heterogeneity nature of lung adenocarcinoma. Some of these methylation aberrations might be cancer subtype specific and associated with distinct clinical outcomes. As a case study, we identified potential methylation aberration signatures correlated with the prognosis of stage I lung adenocarcinoma patients after surgical resection using all the 120 TCGA samples of stage I lung adenocarcinoma patients with surgical resection. For each CpG site, we classified the patients into two groups according to whether they had or had not the methylation aberration of this CpG site and then compared their relapse-free survival times after surgical resection (FDR < 0.1, univariate Cox model). Finally, we found seven genes (ALX4, PDLIM1, FST, EDIL3, ITPKB, APC and UCN) that were significantly associated with the prognosis of patients. For instance, we found that APC was hypermethylated in 50% of the 120 patients and these patients had significantly shorter relapse-free survival time (*p* < 0.0014, univariate Cox model) than the other patients without APC hypermethylation. This result supported a previous report that hypermethylation of APC could be associated with poor prognosis of NSCLC including lung adenocarcinoma [35]. Moreover, ALX4 [36], PDLIM1 [37], CAP2 [38] and EDIL3 [39] have also been found to be correlated with the prognosis of cancer. Thus, the ability of these methylation aberrations for predicting the prognosis of stage I lung adenocarcinoma patients after surgical resection deserves future studies.

In this paper, we only analyzed the CpG sites within gene promoter regions, assayed by both the 27K and 450K arrays, but were unable to analyze the huge number of CpG sites located within gene bodies or intergenic regions due to the extensive computational burden. Thus, an optimized algorithm needs to be developed so that the genome-wide CpG sites assayed by 450K or whole-genome bisulfite sequencing could be taken into account. In addition, in order to extend the application scope of the RankComp algorithm, it is necessary to further evaluate the cross-platform properties of RMOs in samples measured by other platforms such as the whole-genome bisulfite sequencing platform.

In summary, the individual-level analysis of DM CpG sites can help us identifying universal or subtype-specific DNA methylation biomarker for cancer prevention, treatment and diagnosis [40, 41].

## Conclusions

Using multiple DNA methylation datasets for normal lung tissues, we firstly revealed that the RMOs of CpG sites within different samples of normal lung tissues are highly stable but widely reversed in the corresponding cancer tissues. This biological phenomenon allows us to exploit the within-sample RMOs of CpG sites to accurately detect individual-level DM CpG sites using RankComp. Additionally, we used RankComp to identify DM CpG sites for each of the 539 lung adenocarcinoma samples from TCGA and identified many common DNA methylation aberrations in lung adenocarcinoma tissues, which were validated by paired cancer-normal samples. Using gene expression analysis, we further identified abnormal expression genes among genes with common DNA methylation aberrations. These common genes might be used as candidate biomarkers for lung adenocarcinoma diagnosis and therapy.

## Additional files

**Additional file 1: Table S1.** Stable and reversal CpG site pairs identified in the samples measured by two platforms.

**Additional file 2: Table S2.** Consistency of DM CpG sites identified in two independent datasets for lung adenocarcinoma.

**Additional file 3: Table S3.** The frequencies of DM CpG sites in the 539 lung adenocarcinoma samples from TCGA.

**Additional file 4: Table S4.** DNA methylation datasets of paired cancer-normal lung tissues used to validate the hypermethylated and hypomethylated CpG sites with high frequencies.

**Additional file 5: Table S5.** The hypomethylation frequencies of 44 CpG sites in disease samples.

**Additional file 6: Table S6.** Gene expression datasets with paired cancer-normal lung tissues.

**Additional file 7: Figure S1.** The KEGG pathways separately enriched with hypermethylated (a) and hypomethylated (b) genes in at least 10% of the 539 TCGA lung adenocarcinoma samples.

## Abbreviations

DM: differentially methylated
RMOs: relative methylation-level orderings
TCGA: The Cancer Genome Atlas
27K array: Illumina Human Methylation 27 Beadchip
450K array: Illumina Human Methylation 450 Beadchip
KEGG: Kyoto Encyclopedia of Genes and Genomes
FDR: false discovery rate
