# Supplementary material for: Individualized analysis reveals CpG sites with methylation aberrations in almost all lung adenocarcinoma tissues
Source: J Transl Med. 2017 Feb 8;15:26. doi: 10.1186/s12967-017-1122-y (PMC5299650; doi:10.1186/s12967-017-1122-y)
Supplement: Supplementary file 1 — Additional file 1: Table S1. Stable and reversal CpG site pairs identified in the samples measured by two platforms. [file 12967_2017_1122_MOESM1_ESM.doc]

**Table S1.** Stable and reversal CpG site pairs identified in the samples measured by two platforms.

| CpG pair | Tissue | Num_27K | Num_450K | Con_overlap | Con_score |
| --- | --- | --- | --- | --- | --- |
| Stable | Lung | 229,037,151 | 173,949,484 | 157,635,058 | 99.75% |
| Reversal | Lung | 8,615,527 | 37,815,005 | 6,310,115 | 99.79% |

Note: Num_27K and Num_450K represent the numbers of stable (or reversal) CpG site pairs identified in the data assayed by 27K and 450K, respectively. Con_overlap represents concordant overlaps. Con_score represents the concordance score.
