# Supplementary material for: Individualized analysis reveals CpG sites with methylation aberrations in almost all lung adenocarcinoma tissues
Source: J Transl Med. 2017 Feb 8;15:26. doi: 10.1186/s12967-017-1122-y (PMC5299650; doi:10.1186/s12967-017-1122-y)
Supplement: Supplementary file 2 — Additional file 2: Table S2. Consistency of DM CpG sites identified in two independent datasets for lung adenocarcinoma. [file 12967_2017_1122_MOESM2_ESM.doc]

**Table S2.** Consistency of DM CpG sites identified in two independent datasets for lung adenocarcinoma.

| Dataset | Num_hyper | Num_hypo | Con_ hyper | Con_hypo | Con_score |
| --- | --- | --- | --- | --- | --- |
| GSE32866 | 2783 | 2668 | 2363 | 1796 | 100% |
| GSE62948 | 3603 | 2912 |

Num_hyper and Num_hypo represent the numbers of hypermethylated and hypomethylated CpG sites, respectively. Con_hyper and Con_hypo represent the numbers of concordant hypermethylated and hypomethylated CpG sites in two datasets. Con_score is the concordance score.
