# Supplementary material for: Individualized analysis reveals CpG sites with methylation aberrations in almost all lung adenocarcinoma tissues
Source: J Transl Med. 2017 Feb 8;15:26. doi: 10.1186/s12967-017-1122-y (PMC5299650; doi:10.1186/s12967-017-1122-y)
Supplement: Supplementary file 3 — Additional file 3: Table S3. The frequencies of DM CpG sites in the 539 lung adenocarcinoma samples from TCGA. [file 12967_2017_1122_MOESM3_ESM.doc]

**Table S3.** The frequencies of DM CpG sites in the 539 lung adenocarcinoma samples from TCGA.

| Hypermethylated CpG sites | | | | | | | | | | |
| --- | --- | --- | --- | --- | --- | --- | --- | --- | --- | --- |
| CpG sites | Gene symble | Frequency | |  | | CpG sites | | Gene symble | Frequency | |
| cg05050341 | ENG | 92.58% | |  | | cg19797376 | | TAL1 | 90.35% | |
| cg12111714 | ATP8A2 | 91.84% | |  | | cg26521404 | | HOXA9 | 91.28% | |
| cg19466563 | SPARCL1 | 92.39% | |  | |  | |  |  | |
| Hypomethylated CpG sites | | | | | | | | | | |
| CpG site | Gene symble | Frequency |  | | CpG site | | Gene symble | | Frequency | |
| | cg00411097 | | --- | | cg00918005 | | cg01656853 | | cg02131853 | | cg02868123 | | cg03421300 | | cg04837071 | | cg04947157 | | cg04956511 | | cg05440289 | | cg06123346 | | cg08214029 | | cg08314660 | | cg08475088 | | cg08886154 | | cg09325711 | | cg09924998 | | cg11204562 | | cg11802013 | | cg13179915 | | cg13797031 | | cg13897627 | | | MGC9712 | | --- | | REG3G | | FUT2 | | FLJ23235 | | REGL | | SFN | | NOXA1 | | TMC6 | | PTPN6 | | IVL | | ATP4A | | CCL18 | | PKP3 | | NALP9 | | PAX4 | | RALA | | VPS33A | | C10orf81 | | CCND1 | | KCNK7 | | NIPSNAP1 | | FLJ44674 | | | 92.02% | | --- | | 90.91% | | 90.54% | | 91.28% | | 92.02% | | 91.65% | | 90.17% | | 95.92% | | 91.09% | | 91.09% | | 90.72% | | 90.35% | | 94.25% | | 91.28% | | 92.39% | | 90.54% | | 92.02% | | 92.02% | | 93.32% | | 91.09% | | 91.47% | | 91.84% | |  | | | cg14444710 | | --- | | cg14547335 | | cg15387123 | | cg15422147 | | cg15439078 | | cg15633390 | | cg18692273 | | cg18888403 | | cg20828084 | | cg21604615 | | cg21747271 | | cg22346765 | | cg22658979 | | cg22862656 | | cg23696949 | | cg24272559 | | cg24423088 | | cg24898753 | | cg25612480 | | cg26090660 | | cg26530341 | | cg26767897 | | | | PDPK1 | | --- | | ATP2B2 | | CLIC3 | | SERPIN5 | | MYL3 | | EIF4E | | TNNT2 | | HMGCL | | KIAA1199 | | SYTL1 | | AIP | | UNC5CL | | MMP13 | | SCGB2A2 | | LAMC2 | | LOC116123 | | KRTAP8-1 | | FTH1 | | UBE2V2 | | GDEP | | TNFRSF1A | | XDH | | | | | 93.69% | | --- | | 91.28% | | 92.21% | | 90.72% | | 93.69% | | 91.28% | | 91.09% | | 90.54% | | 91.28% | | 91.09% | | 90.72% | | 94.81% | | 93.88% | | 90.54% | | 91.84% | | 91.65% | | 94.99% | | 90.54% | | 91.28% | | 90.54% | | 91.09% | | 93.88% | |
