# Supplementary material for: Individualized analysis reveals CpG sites with methylation aberrations in almost all lung adenocarcinoma tissues
Source: J Transl Med. 2017 Feb 8;15:26. doi: 10.1186/s12967-017-1122-y (PMC5299650; doi:10.1186/s12967-017-1122-y)
Supplement: Supplementary file 4 — Additional file 4: Table S4. DNA methylation datasets of paired cancer-normal lung tissues used to validate the hypermethylated and hypomethylated CpG sites with high frequencies. [file 12967_2017_1122_MOESM4_ESM.doc]

**Table S4.** DNA methylation datasets of paired cancer-normal lung tissues used to validate the hypermethylated and hypomethylated CpG sites with high frequencies.

| Dataset | Number of paired cancer-normal samples | Platform |
| --- | --- | --- |
| GSE62948 | 28 | 27K |
| TCGA | 53 | 27K+450K |
| GSE32861 | 59 | 27K |
| Pair8 | 8 | 450K |

Pair8 represents the eight paired cancer-normal lung samples detected by us.
