# Supplementary material for: Individualized analysis reveals CpG sites with methylation aberrations in almost all lung adenocarcinoma tissues
Source: J Transl Med. 2017 Feb 8;15:26. doi: 10.1186/s12967-017-1122-y (PMC5299650; doi:10.1186/s12967-017-1122-y)
Supplement: Supplementary file 5 — Additional file 5: Table S5. The hypomethylation frequencies of 44 CpG sites in disease samples. [file 12967_2017_1122_MOESM5_ESM.doc]

**Table S5.** The hypomethylation frequencies of 44 CpG sites in disease samples.

| CpG site | Frequency_1 | Frequency_2 |  | CpG site | Frequency_1 | Frequency_2 |
| --- | --- | --- | --- | --- | --- | --- |
| | cg00411097 | | --- | | cg00918005 | | cg01656853 | | cg02131853 | | cg02868123 | | cg03421300 | | cg04837071 | | cg04947157 | | cg04956511 | | cg05440289 | | cg06123346 | | cg08214029 | | cg08314660 | | cg08475088 | | cg08886154 | | cg09325711 | | cg09924998 | | cg11204562 | | cg11802013 | | cg13179915 | | cg13797031 | | cg13897627 | | | 99.29% | | --- | | 96.43% | | 97.86% | | 97.14% | | 94.29% | | 96.43% | | 97.14% | | 99.29% | | 97.86% | | 91.43% | | 98.57% | | 95.71% | | 96.43% | | 90.00% | | 98.57% | | 96.43% | | 97.14% | | 96.43% | | 93.57% | | 91.43% | | 95.00% | | 95.71% | | | 100.00% | | --- | | 100.00% | | 100.00% | | 100.00% | | 100.00% | | 87.50% | | 100.00% | | 100.00% | | 100.00% | | 100.00% | | 87.50% | | 100.00% | | 100.00% | | 100.00% | | 100.00% | | 100.00% | | 100.00% | | 100.00% | | 37.50% | | 75.00% | | 100.00% | | 100.00% | |  | | cg14444710 | | --- | | cg14547335 | | cg15387123 | | cg15422147 | | cg15439078 | | cg15633390 | | cg18692273 | | cg18888403 | | cg20828084 | | cg21604615 | | cg21747271 | | cg22346765 | | cg22658979 | | cg22862656 | | cg23696949 | | cg24272559 | | cg24423088 | | cg24898753 | | cg25612480 | | cg26090660 | | cg26530341 | | cg26767897 | | | 98.57% | | --- | | 91.43% | | 99.29% | | 95.71% | | 92.14% | | 97.86% | | 87.86% | | 95.71% | | 95.71% | | 99.29% | | 95.71% | | 97.14% | | 94.29% | | 90.71% | | 98.57% | | 94.29% | | 97.86% | | 98.57% | | 96.43% | | 93.57% | | 97.86% | | 96.43% | | | 87.50% | | --- | | 100.00% | | 100.00% | | 100.00% | | 75.00% | | 100.00% | | 87.50% | | 100.00% | | 100.00% | | 100.00% | | 100.00% | | 100.00% | | 100.00% | | 75.00% | | 87.50% | | 87.50% | | 100.00% | | 100.00% | | 100.00% | | 75.00% | | 100.00% | | 87.50% | |

For a gene corresponding to a CpG site, Frequency^1^ and Frequency^2^ represent the hypomethylation frequencies in the 140 publicly available and eight additionally measured paired cancer-normal samples, resepectively.
