# Supplementary material for: Individualized analysis reveals CpG sites with methylation aberrations in almost all lung adenocarcinoma tissues
Source: J Transl Med. 2017 Feb 8;15:26. doi: 10.1186/s12967-017-1122-y (PMC5299650; doi:10.1186/s12967-017-1122-y)
Supplement: Supplementary file 6 — Additional file 6: Table S6. Gene expression datasets with paired cancer-normal lung tissues. [file 12967_2017_1122_MOESM6_ESM.doc]

**Table S6.** Gene expression datasets with paired cancer-normal lung tissues.

| Dataset | Number of paired  cancer-normal samples | Platform |
| --- | --- | --- |
| GSE32867 | 57 | Illumina HumanWG-6 v3.0 expression beadchip |
| TCGA | 25 | IlluminaHiSeq_RNASeq |
| Pair8 | 8 | Human Gene Expression Microarray V4.0 |

Pair8 represents the eight paired cancer-normal lung samples detected by us.
