# Supplementary figures and images for: Individualized analysis reveals CpG sites with methylation aberrations in almost all lung adenocarcinoma tissues
Source: J Transl Med. 2017 Feb 8;15:26. doi: 10.1186/s12967-017-1122-y (PMC5299650; doi:10.1186/s12967-017-1122-y)

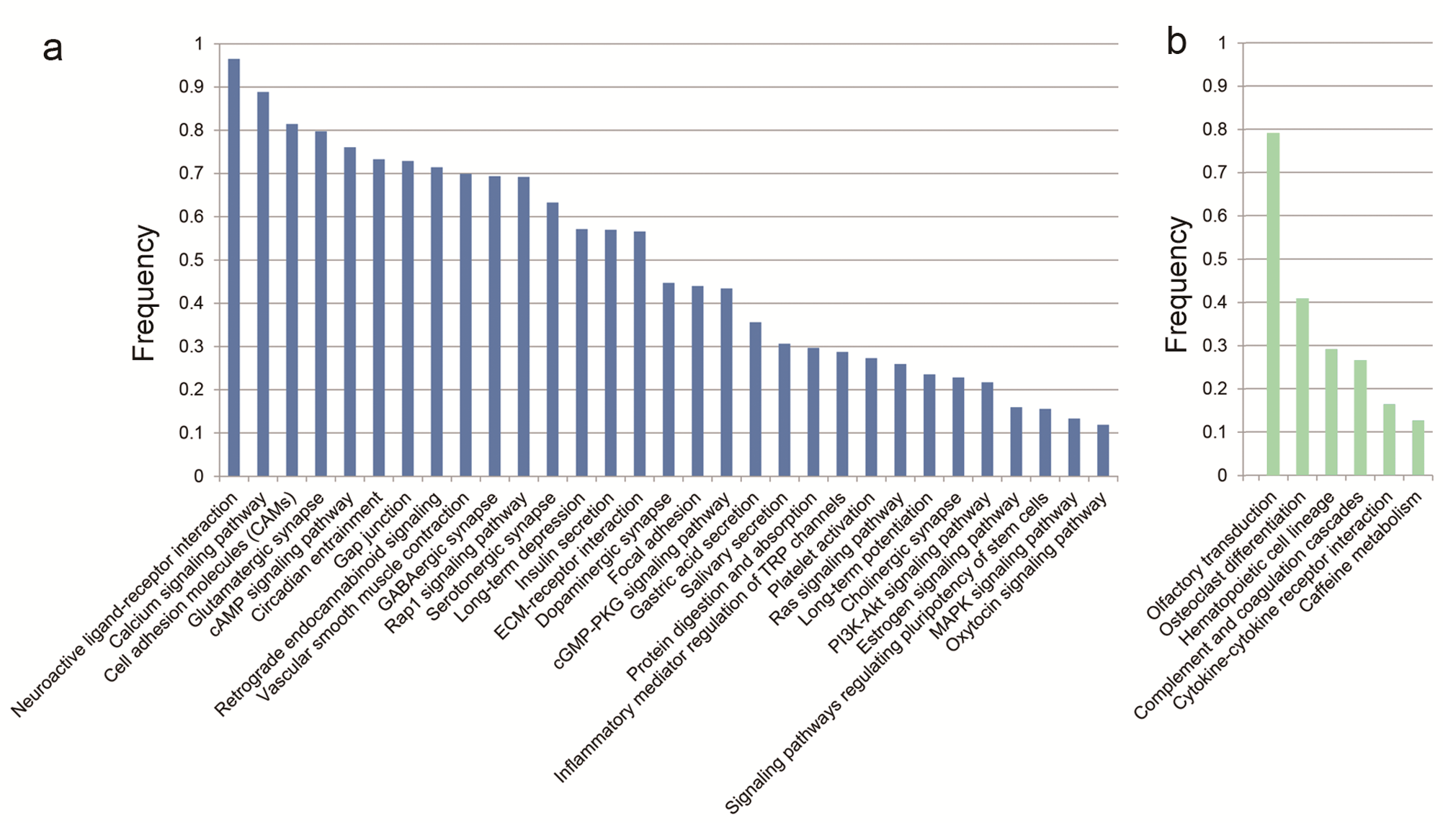

Supplement: Supplementary file 7 — Additional file 7: Figure S1. The KEGG pathways separately enriched with hypermethylated (a) and hypomethylated (b) genes in at least 10% of the 539 TCGA lung adenocarcinoma samples. [file 12967_2017_1122_MOESM7_ESM.tif]
